# Facilitating the Implementation Process of High-Performance Work Systems: The Role of Authentic Leadership

**DOI:** 10.3389/fpsyg.2020.550711

**Published:** 2020-09-25

**Authors:** Man Cao, Yixuan Zhao, Shuming Zhao

**Affiliations:** School of Business, Nanjing University, Nanjing, China

**Keywords:** high-performance work systems, authentic leadership, commitment-focused HR attributions, well-being-focused HR attributions, performance-focused HR attributions, thriving at work

## Abstract

Although substantive research has devoted increasing attention to variability in human resource practices at the organization, group, and individual levels, the critical role of line managers’ leadership in predicting this variability in the human resource management delivery and implementation process has been overlooked. Drawing from social information processing theory and human resource (HR) attributions theory, this study proposes that authentic leadership moderates the positive relationship between department-level high-performance work systems and employee-perceived high-performance work systems. Moreover, employee-perceived high-performance work systems can enhance employees’ thriving at work through commitment-focused HR attributions (well-being and performance). Analyzing the matched data from 145 departments and 834 employees, we found that the extent to which department-level high-performance work systems are positively related to employee-perceived high-performance work systems depends on authentic leadership within departments. We also found that employee-perceived high-performance work systems will result in commitment-focused HR attributions (well-being and performance), which in turn motivate employees to thrive at work. This study sheds light on whether and how line managers’ leadership influences the HR management process.

## Introduction

Since the 1990s, the accumulated evidence has demonstrated the potential benefits of high-performance work systems (HPWS) (Huselid, 1995; Becker and Gerhart, 1996; Collins and Smith, 2006), which refer to a series of independent and interconnected HR practices designed to enhance employees’ skills and motivations (Huselid, 1995; Datta et al., 2005). However, scholars and practitioners have only recently begun to pay more attention to the human resource (HR) process (Bowen and Ostroff, 2004; Ostroff and Bowen, 2016), referring to how HR systems are delivered and implemented across organizational hierarchies (Ostroff and Bowen, 2016). Indeed, some empirical inquiries have shown that there exists a significant difference between the implemented HPWS and employee-perceived HPWS (Liao et al., 2009). As a result, explicating how to implement HPWS in line with those perceived becomes prominent both theoretically and practically.

To this end, several scholars have provided valuable insights into this issue. For example, Nishii and Wright (2007) extended the typical model in the strategic human resource management area, which suggests that well-implemented HR systems will autonomously contribute to desirable outcomes. They further posited that variability exists within organizations, which constitutes a complexity that requires more scholarly attention. In this vein, Liao et al. (2009), through empirical testing, found that there was a divergence between managers’ perspective on HPWS and employee-perceived HPWS. However, the variance between implemented HPWS and employees’ perceptions has been underexplained, and the pivotal role of line managers in predicting it has been overlooked (Nishii and Paluch, 2018; Kehoe and Han, 2020). Given that line managers often act as the intermediate linkage between intended HR function and employees and the proximal and direct resources for employees to gain information about HR practices, our primary purpose is to explore how line managers influence the alignment of HPWS.

To unveil how line managers shape the implementation process of HPWS, the leadership literature may provide a novel lens, as leadership captures a significant effect on employees’ sensemaking process (Schein, 1992). In general, line managers often enact HPWS and are responsible for translating formal practices into daily interactions with employees (Nishii and Wright, 2007; Vermeeren, 2014). Therefore, the present study draws upon social information processing theory and argues that the alignment of the implementation process of HPWS is likely to be contingent on line managers’ authentic leadership, which is defined as “a pattern of leader behavior that draws upon and promotes both positive psychological capacities and a positive ethical climate to foster greater self-awareness, an internalized moral perspective, balanced processing of information, and relational transparency on the part of leaders working with followers, fostering positive self-development” (Walumbwa et al., 2008). There are several theoretical reasons for which we focus on authentic leadership in the present study. On the one hand, authentic leadership has been seen as the best aligned with HPWS in both the “self-enhancement” and “openness to change” dimensions with regard to their value-based influence on employees (Leroy et al., 2018). Thus, HPWS and authentic leadership may interact and form a better dynamic fit to make employees motivated in organizations (Gill et al., 2018). On the other hand, authentic leaders not only possess internal capabilities, such as leader integrity, which contribute to effectively enacting HPWS but can also enable employees to perceive the “legitimacy, credibility, and authenticity” of HPWS while continuously displaying their inherent attractiveness (Gill et al., 2018). Thus, we propose that the authentic leadership of line managers would strengthen the relationship between the actual HPWS and the employee-perceived HPWS.

Furthermore, although the HR process began to be highlighted in strategic human resource management (SHRM) research since Bowen and Ostroff (2004) put forward their initial framework, few studies thoroughly examine the whole influence chain of actual HR practices, that is, those enacted by line managers, perceived by employees, subjectively interpreted and finally influencing employee attitudes or behaviors. Therefore, after examining the moderating role of authentic leadership in the relationship between department-level HPWS and employee-perceived HPWS, we draw upon HR attribution theory and examine how employee-perceived HPWS elicit employees’ HR attributions, which in turn contribute to thriving at work.

Whereas prior research has documented that perceived HPWS are positively associated with favorable attitudes and behaviors, most of these works are based on social exchange theory or the ability, motivation, and opportunity model (Takeuchi et al., 2007; Gong et al., 2010). However, according to HR attribution theory (Nishii et al., 2008), the effect of employee-perceived HPWS on attitudes or behavioral outcomes may be more complex rather than straightforward because employees often make interpretations and relevant attributions for events or others’ behaviors (Heider, 1958; Hewett et al., 2018), which have a significant effect on their subsequent attitudes and behaviors. Thus, we go one step further and examine how the HR attributions derived from employee-perceived HPWS influence their sense of thriving at work.

The present study makes several key contributions to both the SHRM and the leadership literature. First, our study advances the HRM literature by integrating HPWS with the leadership literature and exploring the critical role of line managers, especially authentic leadership, in the implementation of HPWS. Answering the call of Ostroff and Bowen (2016) to examine the role of leaders in facilitating strong HR systems within units or organizations, we posit that the relationship between department-level HPWS and employee-perceived HPWS will be pronounced when line managers are authentic leaders. Second, the present research integrates social information processing theory and HR attributions theory to examine how HPWS are implemented by line managers and affects employees’ thriving at work, which provides a nuanced understanding of the whole causal chain of the HR process. Third, we extend the nomologic network of thriving at work by identifying HPWS and commitment-focused HR attributions as antecedents.

**FIGURE 1:**
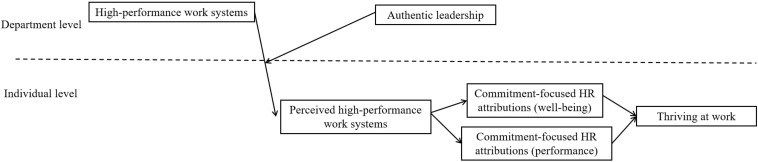
Conceptual model.

Figure 1 depicts our theoretical model.

## Theoretical Background and Hypotheses

### Moderating Role of Authentic Leadership

High-performance work systems are defined as a series of independent and interconnected HR practices designed to enhance employees’ skills, motivation, and opportunities and ultimately improve organizational performance (Huselid, 1995; Sun et al., 2007; Takeuchi et al., 2007). In general, HPWS involves multiple HR practices such as selective staffing, extensive training, incentive-based compensation, decision-making participation, and information sharing (Takeuchi et al., 2007; Aryee et al., 2012). The implementation process of HPWS suggests that organizations’ actual HR practices would be perceived by employees through communications between organizations and employees. These employees’ perceptions of HPWS will, in turn, result in individual reactions, such as attitudes and behaviors (Nishii and Wright, 2007).

Although HPWS is seen as organizational politics, HR implementations within teams and groups often require line managers to translate the organizational practices into situation-specific actions (Nishii and Wright, 2007; Zohar, 2000). Specifically, managers may have explicit and implicit discretion when they implement these practices, as organizations may not capture all the contingencies regarding implementation processes. As a result, different HPWS perceptions of employees at the team level emerge. Thus, variability existing at the department level is mostly a function of how line managers, after formal notification, implement HR practices (Purcell and Hutchinson, 2007; Nishii and Wright, 2007; Sikora and Ferris, 2014).

Furthermore, line managers can affect employees’ perceptions of HPWS through their leadership behaviors (Daniel, 1985), which demonstrates the capabilities and willingness of line managers to implement HR practices. As Gill et al. (2018) noted, authentic leadership not only inherently possesses multiple positive capabilities, such as sharing information openly and maintaining their integrity, but also effectively handles various demands of organizational stakeholders in the complex enactment environment (Avolio and Gardner, 2005), which may facilitate the alignment of HPWS in the implementation process. Therefore, we focus on authentic leadership and argue that it can enhance the alignment between department-level HPWS and employee-perceived HPWS.

Social information processing theory provides a theoretical rationale for explicating the moderating role of authentic leadership in the relationship between department-level HPWS and employee-perceived HPWS. The core tenant of social information processing theory is that social information clues help individuals interpret and then construct the context, which subsequently influences their attitudes and behaviors (Salancik and Pfeffer, 1978). In line with this theory, line managers’ behaviors, especially authentic leadership, tend to be seen as a critical source for employees to recognize how they behave appropriately (Lord and Maher, 2002; Lau and Liden, 2008), as line managers typically have the definitive authority in regard to rewards and punishments within departments (French and Raven, 1959; Mawritz et al., 2012).

Specifically, authentic leadership includes multiple inherent positive capabilities (Luthans and Avolio, 2003; Avolio et al., 2004), such as leaders expressing their true self to others and behaving with their internalized moral perspective (Shamir and Eilam, 2005). Due to these prominent characteristics of line managers, it is instructive to enhance the sense of credibility, integrity, and authenticity of HR practices within departments (Gill et al., 2018), which in turn affects the degree of employees’ acceptance and, ultimately, the effectiveness of HPWS (Purcell et al., 2003).

Also, authentic leaders emphasize relational transparency and balance processing (Walumbwa et al., 2008) and respect different views of employees about HPWS. This not only allows authentic leaders to make the implementation style of HR practices better suit the specific situations of departments (Khilji and Wang, 2006) but also creates an open environment for employees to accurately understand HR practices through these transparent interactions. As individual differences exist among employees, may have different views and perspectives regarding HPWS. When authentic leaders provide a two-way open dialog for followers (Gill et al., 2018), employees are likely to gain more relevant information about HPWS and perceive the actual HPWS more accurately.

Accordingly, we suggest that authentic leadership facilitates the alignment between department-level HPWS and employee-perceived HPWS.

Hypothesis 1: Authentic leadership moderates the positive relationship between department-level high-performance work systems and employee-perceived high-performance work systems.

### Employee-Perceived High-Performance Work Systems, Commitment-Focused Human Resource Attributions (Well-Being and Performance), and Thriving at Work

To provide further insight into the implementation process of HPWS, we tend to illuminate how employees perceive and interpret these HR practices, thereby having a consequential effect on their feelings of thriving at work. According to the HR attributions theory (Nishii et al., 2008), employees make causal explanations for the intent of HR practices, and these attributions can subsequently affect attitudes and behaviors. As the prior attribution theory literature (Heider, 1958; Koys, 1988, 1991) and the initial theorizing of HR attributions have noted, HR attributions can be categorized by internal and external attributions. Internal HR attributions refer to the perceptions that the reason for which HR practices are implemented is organizations’ autonomous choice, whereas external attributions exist when HR practices are perceived just as the conformity of external constraints (Nishii et al., 2008; Hewett et al., 2018). Because internal attributions have a more profound effect on employees’ attitudes and behaviors, the present research focuses on one specific internal attribution, commitment-focused HR attributions (well-being and performance), which capture the intended purpose of HR practices: to enhance employees’ well-being and help encourage increased performance to achieve organizational goals (Nishii et al., 2008; Hewett et al., 2019).

We predict that employee-perceived HPWS are likely to elicit commitment-focused HR attributions (well-being and performance).

Commitment-Focused Human Resource Attributions (Well-Being): It is well established that HR practices, particularly HPWS, can function as “signals” or expected intentions of organizations (Ostroff and Bowen, 2000; Kooij et al., 2010). Specifically, HPWS, including extensive training, participation in decision-making, incentive compensation, and so on, connote organizations’ long-term investment for employees (Sun et al., 2007), which also signals that the belief underlying HPWS lies in individual development rather than exploitation. For example, Heffernan and Dundon (2016) argue that HR practices can have a signaling effect on employees by increasing their feelings of value and worth. Furthermore, HPWS can provide more psychological resources and help employees better deal with job demands (Agarwal and Farndale, 2017), which in turn elicit employees’ sense of organizational support and engender their well-being attributions.

Commitment-Focused Human Resource Attributions (Performance): Along with commitment-focused HR attributions (well-being), we also posit that employee-perceived HPWS are positively related to commitment-focused HR attributions (performance). In line with the SHRM literature (Huselid, 1995; Becker et al., 1998), the primary goal of HPWS is to promote individual performance and ultimately, organizational performance by enhancing employees’ skills, motivations, and opportunities. Moreover, consistent with the resource-based review (Becker, 1964; Delery, 1998), HPWS respect employees’ unique values (Walton, 1985) and treat them as important assets of organizations rather than a cost that needs to be reduced, which signals to employees that HR practices aim to promote the enhancement of performance to gain organizational goal attainment.

Accordingly, we hypothesize the following:

Hypothesis 2a: Employee-perceived high-performance work systems are positively related to well-being-focused HR attributions.

Hypothesis 2b: Employee-perceived high-performance work systems are positively related to performance-focused HR attributions.

Next, we suggest that well-being-focused HR attributions will be linked to thriving at work. Thriving at work is defined as a psychological state that contains joint feelings of vitality and learning (Spreitzer et al., 2005). When employees gain more sense that HPWS are intended to enhance well-being, a relevant safe and favorable environment is provided for employees to engage in agentic behaviors (Spreitzer et al., 2005; Jiang et al., 2019), which have been widely seen as the precursor to thriving at work (Niessen et al., 2012; Paterson et al., 2014). Put differently, when feeling more caring from management, employees are likely to be pleasurable at work (Kleine et al., 2019) and motivated to perform exploration and experimentation autonomously in that context, which increases the likelihood that they will gain the experience of personal growth. Additionally, employees tend to develop heedful relating with supervisors and coworkers within these organizations (Porath et al., 2012; Paterson et al., 2014) and, in turn, feel more energized and gain more knowledge or skills during daily communications.

In addition, when employees attribute HPWS to the enhancement of individual performance and ultimately organizational performance, the explicit link between individual work and business strategy fosters their sense of positive meaning, which comes from “the creation of worth or value with work, colleagues, or the organization” (Spreitzer et al., 2005). In these situations, these employees are likely to be task-focused (Brown and Ryan, 2003), devote their full attention to their own work, and immerse themselves in daily activities. Consequently, engaged employees should perceive a heightened level of vitality and acquire more knowledge to help organizations achieve their goals.

In sum, we posit the following:

Hypothesis 3a: Well-being-focused human resource attributions mediate the positive relationship between employee-perceived high-performance work systems and thriving at work.

Hypothesis 3b: Performance-focused human resource attributions mediate the positive relationship between employee-perceived high-performance work systems and thriving at work.

### Serial Mediated Moderation Effects

As the discussion mentioned earlier noted, authentic leadership enables the alignment between department-level HPWS and employee-perceived HPWS by demonstrating the credibility, integrity, and authenticity of HR practices and better utilizing their abilities in the HR implementation process (Avolio and Gardner, 2005; Gill et al., 2018). Because of the positive effects resulting from authentic leaders’ behaviors, followers are likely to form perceptions of an integrated HPWS. When individuals perceive HR practices, they tend to inherently make specific attributions for these practices (Nishii et al., 2008; Hewett et al., 2019), which subsequently impact their attitudes and behaviors. As HPWS demonstrates, organizations not only care about employees’ well-being but also emphasize the significance of performance; employees are likely to foster well-being-focused HR attributions and performance-focused HR attributions. When employees treat HPWS as organizational practices aiming to enhance their well-being (well-being-focused HR attributions), they will feel safer and freer to conduct agentic behaviors (Spreitzer et al., 2005; Jiang et al., 2019), which fuel their feelings of thriving at work. Also, when employees recognize that HPWS are designed to enhance their performance and, in turn, contribute to organizational performance (performance-focused HR attributions), the positive association between their contributions and business strategy may enable employees to gain more meaningful personal experience at work (Brown and Ryan, 2003; Spreitzer et al., 2005), which subsequently facilitates employees’ thriving at work.

In line with the process model of HPWS, social information processing theory, and HR attributions theory, we expect that employee-perceived HPWS and commitment-focused HR attributions (well-being-focused and performance-focused HR attributions) mediate the interacting effect of department-level HPWS and authentic leadership on thriving at work. Specifically, authentic leadership and department-level HPWS interact to affect employee-perceived HPWS. Employee-perceived HPWS, in turn, foster commitment-focused HR attributions (well-being and performance), which promote thriving at work. Thus, we hypothesize the following serial mediated moderation effects:

Hypothesis 4a: The interaction effect of department-level authentic leadership and department-level high-performance work systems on thriving at work is through employee-perceived high-performance work systems and well-being-focused human resource attributions.

Hypothesis 4b: The interaction effect of department-level authentic leadership and department-level high-performance work systems on thriving at work is through employee-perceived high-performance work systems and performance-focused human resource attributions.

## Materials and Methods

### Sample and Procedure

We collected data from 99 organizations ranging from manufacturing, high-tech, and service industries in six main provinces of China. Through the alumni network, chief executive officers of these organizations were connected and invited to participate in this study. We first contacted these chief executive officers one by one, explained the purpose of the current study, and asked them to request their HR managers support our data collection. Under the support of human resource managers, questionnaires with unique identification codes and blank envelopes were assigned to department managers and their subordinates to pair the data from two sources. To reduce selection bias, we mentioned on the first page of the questionnaires that the present study aims to better understand HRM in practice and did not enclose the true research purpose to respondents. Additionally, we confirmed data confidentiality and recommended that respondents seal their surveys in blank envelopes by themselves after completing their questionnaires.

To reduce common method bias, this study collected data from two different sources, department managers and employees. Department managers reported department-level high-performance work systems. Employees completed the measures of perceived HPWS, well-being-focused HR attributions, performance-focused HR attributions thriving at work, and authentic leadership. Initially, our sample consisted of 99 organizations, 186 department managers, and 1,182 employees. After removing unpaired data and void response, the final sample included 145 departments and 834 paired employees. The response rates for department managers and employees were 77.9 and 70.6%, respectively. At the employee level, 53% were male, 47% were female, 23.2% were under 35 years old, 32.9% were 36 to 40 years old, 23.7% were 41–45 years old, and 20.2% were above 46 years old. A total of 87.1% had a college degree. The average tenure of employees was 46.7 months.

### Measures

All the measures of the focal variables were adopted from the leading management journals and extensively used in the management area. Following the translation and back-translation procedures recommended by Brislin (1980), we used the following measures.

Department-level High-Performance Work Systems: Department-level HPWS were measured using Fu et al. (2017) 15-item scale ranging from 1 (strongly disagree) to 7 (strongly agree). A sample item is “The organization provides formal performance appraisals from more than one source (i.e., from several individuals such as supervisors, peers, etc.).” The Cronbach’s alpha value was 0.948.

Employee-Perceived High-Performance Work Systems: Employee-perceived HPWS were rated by employees using Fu et al. (2017) 15-item scale ranging from 1 (strongly disagree) to 7 (strongly agree). Sample items include “Receive continuous training, e.g., continuous professional development” and “Have access to company incentive plans, profit-sharing plans, and/or gainsharing plans.” The Cronbach’s alpha value was 0.939.

Commitment-focused Human Resource Attributions (Well-Being and Performance): Commitment-focused HR attributions contain well-being and performance-focused HR attributions (Nishii et al., 2008; Hewett et al., 2019): employees reported five-item measures of well-being-focused HR attributions and five-item measures of performance-focused HR attributions developed by Nishii et al. (2008). A sample item of well-being-focused HR attributions is the following: “The company pays its employees what it does so that employees will feel valued and respected.” The Cronbach’s alpha value of well-being-focused HR attributions was 0.879. The Cronbach’s alpha value of performance-focused HR attributions was 0.896.

Thriving at Work: Thriving at work was measured using 11-item scales adapted by Russo et al. (2018). Sample items include the following: “I feel a lot of excitement when I am doing my work” and “I learn at work enable me to thrive in life.” The Cronbach’s alpha value was 0.933.

Authentic Leadership: Authentic leadership was rated by employees using a 14-item scale developed by Neider and Schriesheim (2011). Sample items include the following: “My department leader carefully listens to alternative perspectives before reaching a conclusion” and “My department leader shows that he/she understands his/her strengths and weaknesses.” The Cronbach’s alpha value was 0.835. In this study, we aggregated employees’ rated authentic leadership to gain department-level authentic leadership. Rwg and intraclass correlation coefficient (ICC) (1) for authentic leadership were calculated to verify the appropriateness for aggregating (James et al., 1984; LeBreton and Senter, 2008). The results showed that the mean and median of Rwg were 0.83 and 0.90, respectively, indicating that there is sufficient agreement of employee responses within departments. ICC (1) was 0.21, demonstrating that authentic leadership between departments accounted for 21% of the total variance.

### Control Variables

We controlled for two-level variables that may affect employees’ thriving at work. Consistent with previous studies (Russo et al., 2018; Cullen et al., 2018), we controlled for employees’ sex, age, education, and tenure at the individual level and controlled for leaders’ sex, age, and education at the department level.

## Results

### Descriptive Statistics and Confirmatory Factor Analysis

Table 1 presents the means, standard deviations, and intercorrelations among all the variables. To verify the distinctiveness of all variables at two levels (i.e., department-level HPWS, employee-perceived HPWS, commitment-focused attributions (well-being and performance), thriving at work, and authentic leadership), we conducted multilevel confirmatory factor analyses using Mplus 7.4. Given that if all the latent variables at two levels are estimated, the ratio of the sample size to the estimated parameters is less than 5 (Bentler and Chou, 1987); we first conducted item parceling to reduce the number of items for each variable following the recommendations of Landis et al. (2000). In line with previous research (Liu et al., 2015; Sherf et al., 2019), this pairing procedure for item parceling combines the items with the largest loading and the smallest loading and combines the items with the second-largest loading and second-smallest loading after conducting exploratory factor analysis for each latent construct. Table 2 presents the multilevel confirmatory factor analysis results. Compared with other alternative models, the hypothesized model that contains six factors: department-level HPWS, employee-perceived HPWS, commitment-focused attributions (well-being and performance), thriving at work, and authentic leadership, showed excellent fit to the data (χ^2^ = 127.590, RMSEA = 0.027, SRMR for within = 0.028, CFI = 0.991, and NNFI = 0.987). This provided support for the construct validity of all the variables in this study.

**TABLE 1:** Descriptive statistics and intercorrelations.

**Variables**	**Mean**	**S.D.**	**1**	**2**	**3**	**4**	**5**	**6**	
**Individual-level**									
(1) Sex	1.47	0.50							
(2) Age	3.57	1.41	–0.037						
(3) Education	2.46	0.83	0.025	−0.167**					
(4) Tenure	46.66	50.68	0.034	0.503**	−0.136*				
(5) Employee perceived HPWS	5.47	1.01	–0.009	−0.113**	0.036	0.064			
(6) Commitment-focused HR attributions (Well-being)	5.68	0.98	0.009	−0.077*	0.051	–0.059	0.641**		
(7) Commitment-focused HR attributions (Performance)	5.81	0.95	0.002	0.015	0.029	0.004	0.523**	0.698**	
(8) Thriving at work	5.95	0.88	0.077*	0.025	0.028	–0.002	0.453**	0.490**	0.429**
**Department-level**									
(1) Sex of leader	1.34	0.47							
(2) Age of leader	3.58	1.48	–0.107						
(3) Education of leader	2.86	1.01	–0.060	–0.039					
(4) High-performance work systems	5.51	1.07	0.041	0.006	0.103				
(5) Authentic leadership	5.53	0.82	0.185*	–0.049	0.114	0.193*			

N = 145 departments. *n* = 834 employees. *p < 0.05, **p < 0.01, ***p < 0.001.

**TABLE 2:** Results of confirmatory factor analyses.

**Model**	**χ ^2^**	**df**	**CFI**	**NNFI**	**RMSEA**	**SRMR**
Hypothesized model	127.590	80	0.991	0.987	0.027	0.028
Alternative Model 1 (combine employee perceived HPWS and well-being-focused HR attributions)	584.961	84	0.901	0.873	0.084	0.073
Alternative Model 2 (combine well-being-focused HR attributions and thriving at work)	862.864	84	0.846	0.802	0.105	0.117
Alternative Model 3 (combine employee perceived HPWS, well-being-focused HR attributions and thriving at work)	1,697.699	87	0.682	0.605	0.148	0.107
Alternative Model 4 (combine all the individual-level variables)	3,536.074	90	0.319	0.183	0.213	0.179

*N = 145 departments. *n* = 834 employees.*

### Hypothesis Testing

In this study, given the nested nature of the data, we used HLM6 to estimate the proposed model. Before testing all the hypotheses, ICC (1) values for individual variables (i.e., employee-perceived HPWS, well-being-focused HR attributions, performance-focused HR attributions and thriving at work) were calculated to identify whether the multilevel analysis was needed. We found that ICC (1) values for employee-perceived HPWS, well-being-focused HR attributions, performance-focused HR attributions and thriving at work are 0.27, 0.15, 0.13 and 0.17, respectively, which exceed the recommended cutoff of 0.12. Next, individual-level variables were group-mean-centered, and department-level variables were grand-mean-centered following the recommendations. For Hypotheses 2 and 3, we used the parameter-based resampling method in R to estimate the indirect effects (Preacher and Selig, 2012).

**FIGURE 2:**
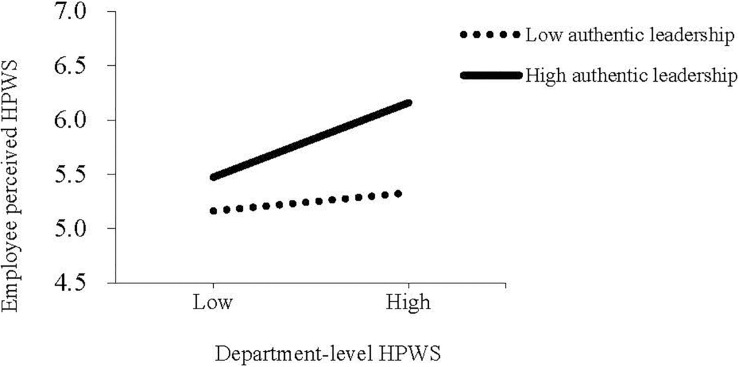
Interaction effect of Department-level HPWS and authentic leadership on employee perceived HPWS.

Hypothesis 1 predicted that authentic leadership moderates the positive relationship between department-level HPWS and employee-perceived HPWS. As model 3 in Table 3 shows, the interaction effect of department-level HPWS and authentic leadership on employee-perceived HPWS was significant (γ = 0.16, *p* < 0.01). Thus, Hypothesis 1 was supported. To clearly demonstrate the moderating role of authentic leadership, we plotted the simple slopes when authentic leadership is at high (+SD) and low (−SD) levels. As Figure 2 shows, when authentic leadership was at a high level, the relationship between department-level HPWS and employee-perceived HPWS was stronger (γ = 0.41, *P* < 0.001). In contrast, when authentic leadership was at a low level, the relationship between department-level HPWS and employee-perceived HPWS was non-significant (γ = 0.10, ns), which also supported Hypothesis 1.

**TABLE 3:** Hierarchical linear modeling results.

**Variables**	**Employee perceived HPWS**			**Well-being-focused HR attributions**		**Performance -focused HR attributions**		**Thriving at work**				
	
	**Mode 1**	**Model 2**	**Model 3**	**Model 4**	**Model 5**	**Model 6**	**Model 7**	**Model 8**	**Model 9**	**Model 10**	**Model 11**	**Model 12**
Intercept	5.27***(0.28)	5.54***(0.28)	5.47***(0.29)	5.07***(0.27)	2.37***(0.41)	5.17***(0.28)	3.33***(0.47)	5.29***(0.26)	5.30***(0.25)	3.27***(0.60)	5.30***(0.25)	3.12***(0.60)
**Individual-level**												
Sex	−0.05(0.08)	−0.05(0.08)	−0.05(0.08)	−0.03(0.06)	−0.00(0.05)	−0.03(0.07)	−0.01(0.06)	0.10(0.06)	0.11*(0.06)	0.12*(0.05)	0.11*(0.05)	0.12*(0.05)
Age	−0.04(0.03)	−0.04(0.03)	−0.03(0.03)	0.01(0.03)	0.01(0.02)	0.03(0.02)	0.04(0.02)	0.03(0.03)	0.03(0.03)	0.05(0.03)	0.03(0.03)	0.04(0.03)
Education	−0.03(0.05)	−0.05(0.05)	−0.05(0.05)	0.05(0.03)	0.03(0.03)	0.02(0.03)	0.01(0.03)	0.04(0.04)	0.03(0.04)	−0.00(0.03)	0.03(0.04)	0.00(0.03)
Tenure	0.00(0.00)	0.00(0.00)	0.00(0.00)	−0.00(0.00)	−0.00(0.00)	0.00(0.00)	0.00(0.00)	−0.00(0.00)	−0.00(0.00)	−0.00(0.00)	−0.00(0.00)	−0.00(0.00)
Perceived HPWS				0.64***(0.04)	0.64***(0.04)	0.56***(0.04)	0.56***(0.04)	0.42***(0.05)	0.26***(0.07)	0.26***(0.07)	0.32***(0.06)	0.32***(0.06)
Well-being-focused HR attributions			−						0.24***(0.05)	0.24***(0.05)		
Performance-focused HR attributions											0.18***(0.04)	0.18***(0.04)
Thriving at work												
**Department-level**												
Sex of leader	0.19+(0.10)	0.10(0.10)	0.08(0.10)	0.15(0.11)	−0.02(0.07)	0.19+(0.10)	0.03(0.07)	0.11(0.10)	0.11(0.10)	−0.02(0.07)	0.11(0.10)	−0.04(0.07)
Age of leader	0.01(0.03)	−0.00(0.03)	−0.00(0.03)	0.02(0.03)	0.02(0.02)	0.02(0.03)	0.02(0.02)	0.04(0.03)	0.04(0.03)	0.02(0.02)	0.04(0.03)	0.02(0.02)
Education of leader	0.07(0.05)	0.06(0.05)	0.06(0.05)	0.07+(0.04)	0.01(0.03)	0.06(0.05)	−0.00(0.03)	0.01(0.04)	0.01(0.04)	−0.04(0.03)	0.01(0.04)	−0.04(0.03)
Department average perceived HPWS					0.57***(0.06)		0.41***(0.07)			0.09(0.10)		0.15(0.10)
Department average well-being-focused HR attributions										0.34**(0.09)		
Department average performance-focused HR attributions												0.30**(0.11)
HPWS	0.28***(0.06)	0.26***(0.06)	0.26***(0.06)		−0.06(0.04)		−0.00(0.03)			0.05(0.04)		0.03(0.04)
Authentic leadership		0.37***(0.07)	0.35***(0.07)		0.28***(0.06)		0.32***(0.06)			0.24**(0.07)		0.24**(0.07)
HPWS* Authentic leadership			0.16**(0.06)		−0.02(0.05)		−0.11*(0.05)			−0.01(0.04)		0.02(0.04)
*R*^2^	0.12	0.16	0.18	0.26	0.44	0.20	0.34	0.13	0.16	0.32	0.15	0.30

N = 834 (individual-level); 145 (department-level). *p < 0.05, **p < 0.01, ***p < 0.001.

Hypotheses 2a and 2b posit that employee-perceived HPWS are positively related to commitment-focused HR attributions (well-being and performance). Model 4 demonstrated that employee-perceived HPWS were positively related to well-being-focused HR attributions (γ = 0.64, *P* < 0.001), supporting Hypothesis 2a. Model 6 showed that employee-perceived HPWS have a positive relationship with performance-focused HR attributions (γ = 0.56, *P* < 0.001), supporting Hypothesis 2b.

Hypotheses 3a and 3b proposed that commitment-focused HR attributions (well-being and performance) would mediate the positive relationship between employee-perceived HPWS and thriving at work. For Hypothesis 3a, as model 8 in Table 3 shows, employee-perceived HPWS were positively associated with thriving at work (γ = 0.42, *P* < 0.001). Employee-perceived HPWS were positively related to well-being-focused HR attributions (γ = 0.64, *P* < 0.001) (Hypothesis 2a). The results of model 9 showed that when employee-perceived HPWS and well-being-focused HR attributions were added, the relationship between employee-perceived HPWS and thriving at work decreased (γ = 0.26, *P* < 0.001), and the effect of well-being-focused HR attributions and thriving at work was also significant (γ = 0.24, *P* < 0.001). Therefore, Hypothesis 3a was supported. We also used the Monte Carlo method to estimate the 95% confidence interval (CI) for the indirect effect. The results showed that the 95% CI was [0.107, 0.207] and did not include zero, supporting Hypothesis 3a. Similarly, for Hypothesis 3b, model 6 showed that employee-perceived HPWS have a positive relationship with performance-focused HR attributions (γ = 0.56, *P* < 0.001). As model 11 shows, the relationship between employee-perceived HPWS and thriving at work decreased (γ = 0.32, *P* < 0.001), and the effect of performance-focused HR attributions and thriving at work was also significant (γ = 0.18, *P* < 0.001) when employee-perceived HPWS and performance-focused HR attributions were added. The 95% CI for the indirect effect in Hypothesis 3b was [0.061, 0.143], which supported Hypothesis 3b.

Hypotheses 4a and 4b stated that the interaction between department-level HPWS and authentic leadership could affect thriving at work though employee-perceived HPWS and commitment-focused HR attributions (well-being and performance). For Hypothesis 4a, as the indirect effect includes three sequential paths, that is, the interaction effect of department-level HPWS and authentic leadership on employee-perceived HPWS (model 3, γ = 0.16, *p* < 0.01), the effect of department average HPWS on well-being-focused HR attributions (model 5, γ = 0.57, *p* < 0.01), and the effect of department average well-being-focused HR attributions on thriving at work (model 10, γ = 0.34, *p* < 0.01). To examine the mediated moderation effect, we conducted parametric bootstrapping for 20,000 repetitions to assess the 95% CI by using R software. The results showed that the 95% CI was [0.028, 0.032] and did not include zero, which supports Hypothesis 4a. Similarly, the 95% CI of the indirect effect in Hypothesis 3b was [0.018, 0.020], which supports Hypothesis 4b.

## Discussion

Drawing upon social information processing theory and HR attributions theory, we investigate whether and how line managers’ authentic leadership influences the process of HPWS. The present research found that the relationship between department-level HPWS and employee-perceived HPWS will be pronounced when line managers are authentic leaders and perceived HPWS indirectly affects employee thriving at work through commitment-focused attributions (well-being and performance).

### Theoretical Contributions

The present study makes several key contributions to the SHRM and leadership literature.

First, our study offers a standpoint to take line managers’ leadership into account to unravel the implementation process of HPWS. It is widely acknowledged that there exists variability between actual HR practices and employees’ perceptions of HR practices (Nishii and Wright, 2007). In this vein, recent studies have considered line managers’ involvement in the HR implementation process to account for that variance (Pak and Kim, 2018). However, most of them predominately focused on the antecedents and consequences of line managers’ HR implementation behavior (Kehoe and Han, 2020); for instance, organizational culture (Dewettinck and Vroonen, 2017), implementation-focused organizational climate (Sikora and Ferris, 2014), and perceived HR support (Dewettinck and Vroonen, 2017) have been positively linked to line managers’ HR implementation behaviors. Little attention has been paid to line managers’ leadership that may function as a medium through which these managers would better enable employees to understand HR practices in the direct influential process. Thus, our study answers Ostroff and Bowen’ (2016) call for examining the role of line managers in facilitating strong HR systems and finds that authentic leadership strengthens the relationship between department-level HPWS and employee-perceived HPWS.

Second, by integrating authentic leadership and HPWS implementation, this study also extends the leadership literature and suggests that authentic leadership and HPWS may interact to affect individual outcomes. In Nishii and Paluch (2018), leadership is not only one style of individuals exerting their influence among teams or organizations but also saturated in a wider environment that contains various interacting factors, such as HR practices. Additionally, due to the similar purpose underlying leadership and HR practices to effectively manage or influence employees, Leroy et al. (2018) recommended future research to deeply investigate the interaction models by which leadership and HR practices may work together (Agarwal and Farndale, 2017). Hence, this study provides a nuanced picture of how line managers’ leadership and HR systems exert their complex effects.

Third, we contribute to the SHRM literature on the HR process by illuminating the subsequent process through which employee-perceived HPWS affect thriving at work. Although recent studies have begun to shift their focus from HR content to HR process, few studies uncover and examine the whole causal chain of HR implementation process that includes enacting HR practices by line managers, receiving and interpretation of these by employees, and, subsequently, how HR practices influence employees’ attitudes. Accordingly, we addressed this question and developed a theoretical model to test the relationships among department-level HPWS, employee-perceived HPWS, employee-commitment-focused HR attributions, and thriving at work. Thus, a comprehensive understanding of the HR process would not only shed light on the contingency perspective but also enrich the SHRM literature.

Finally, this paper adds novel knowledge to the thriving at work literature by identifying HR practices and commitment-focused HR attributions as antecedents of thriving at work. As pointed out by Spreitzer et al. (2005), thriving at work is socially embedded in contexts such as decision-making discretion and information sharing. Considering a broader organizational context, we theorized how HPWS contribute to thriving at work though commitment-focused HR attributions (well-being and performance). Therefore, this study advances the literature on thriving at work and provides a promising pathway to examine the mechanisms underlying it.

### Practical Implications

Our study offers several practical implications for managers. As shown by our results, in the HR implementation process, authentic leadership of line managers does matter in the alignment of HPWS across organizational hierarchies. Thus, organizations should not only pay attention to line managers but also provide more opportunities for them to cultivate these specific leadership styles, which can facilitate organizational goal achievement. There have been studies focusing on the antecedents of authentic leadership; for example, Peus et al. (2012) suggest that when leaders possess more self-knowledge and self-consistency, they are likely to be perceived as authentic leaders.

For HR managers, on the one hand, considering line managers’ actions would benefit monitoring and controlling the effectiveness of HR practices. In addition, unlocking the influence linkage between formal practices and daily behavior manners of line managers is also necessary for the vertical and horizontal fit of HR systems. On the other hand, the implementation of HR systems is a process, and each part of the causal chain may be affected by various contextual factors. Thus, a process perspective is critical for HR managers to capture the complete picture of organizational systems and better manage HR implementation.

### Limitations and Future Research

Although our data are from multiple sources, department managers and employees, there are several limitations to our research. First, as our research design is cross-sectional, the causal inferences among variables cannot be confirmed in this study. Thus, we encourage future studies to use a longitudinal research design to further test this theoretical model. Second, our study only focuses on one specific leadership of line managers, authentic leadership, because of the similar value underlying it and HPWS and the unique contributions of authentic leadership to the alignment of HR implementations. As Leroy et al. (2018) suggested, the interplay of leadership and HR practices would have multiple models, such as dynamic fit, which capture the interaction between these two factors would evolve and change over time (Jansen and Shipp, 2013). Therefore, we call for more research to explore whether and how leadership and HR practices co-affect important outcomes at the organization or team level. Third, although we draw from HR attributions theory and suggest that commitment-focused HR attributions act as the key mechanism between employee-perceived HPWS and thriving at work, our study merely concentrates on internal attributions and does not consider external attributions. Future research is recommended to include other attributions as control variables as well when examining one specific attribution, thus providing a nuanced understanding of the effects. Finally, despite our data samples containing various industries, they are all from China, which may raise the concern of generalizability to other cultural contexts. It is necessary for future studies to further test the theoretical model of this study.

## Data Availability Statement

The datasets presented in this article are not readily available because according to the contracts signed with organizations before collecting data, we have to confirm data confidentiality and cannot make it public. Requests to access the datasets should be directed to MC, njucaoman@126.com.

## Ethics Statement

Ethical review and approval was not required for the study on human participants in accordance with the local legislation and institutional requirements. Written informed consent from the patients/participants or patients/participants legal guardian/next of kin was not required to participate in this study in accordance with the national legislation and the institutional requirements.

## Author Contributions

MC put forward the conceptual model and wrote the manuscript. YZ conducted the language editing and helped to improve the flow of the manuscript. SM collected the data and made valuable suggestions for both the initial draft and subsequent revisions. All authors contributed to the article and approved the submitted version.

## Conflict of Interest

The authors declare that the research was conducted in the absence of any commercial or financial relationships that could be construed as a potential conflict of interest.
